# Novel agents that downregulate EGFR, HER2, and HER3 in parallel

**DOI:** 10.18632/oncotarget.3398

**Published:** 2015-03-19

**Authors:** Renan Barroso Ferreira, Mary Elizabeth Law, Stephan Christopher Jahn, Bradley John Davis, Coy Don Heldermon, Mary Reinhard, Ronald Keith Castellano, Brian Keith Law

**Affiliations:** ^1^ Department of Chemistry, University of Florida, Gainesville, FL 32611, USA; ^2^ Department of Pharmacology & Therapeutics, University of Florida, Gainesville, FL 32610, USA; ^3^ Department of Medicine, University of Florida, Gainesville, FL, 32610, USA; ^4^ Department of Infectious Diseases and Pathology, University of Florida, Gainesville, FL, 32610, USA

**Keywords:** HER2, disulfide bonds, EGFR, HER3, breast cancer

## Abstract

EGFR, HER2, and HER3 contribute to the initiation and progression of human cancers, and are therapeutic targets for monoclonal antibodies and tyrosine kinase inhibitors. An important source of resistance to these agents arises from functional redundancy among EGFR, HER2, and HER3. EGFR family members contain conserved extracellular structures that are stabilized by disulfide bonds. Compounds that disrupt extracellular disulfide bonds could inactivate EGFR, HER2, and HER3 in unison. Here we describe the identification of compounds that kill breast cancer cells that overexpress EGFR or HER2. Cell death parallels downregulation of EGFR, HER2, and HER3. These compounds disrupt disulfide bonds and are termed Disulfide Bond Disrupting Agents (DDAs). DDA RBF3 exhibits anticancer efficacy *in vivo* at 40 mg/kg without evidence of toxicity. DDAs may complement existing EGFR-, HER2-, and HER3-targeted agents that function through alternate mechanisms of action, and combination regimens with these existing drugs may overcome therapeutic resistance.

## INTRODUCTION

The Epidermal Growth Factor Receptor (EGFR) family members EGFR, Human Epidermal growth factor Receptor-2 (HER2), and Human Epidermal growth factor Receptor-3 (HER3) are well established as proto-oncogenes that play key roles in the initiation and progression of human cancers (reviewed in [1–3]). EGFR is frequently mutationally activated in lung cancer and is the target of the FDA-approved drugs Cetuximab, Panitumumab, and Erlotinib. Although EGFR is rarely mutated in breast cancers, the wild type protein is frequently overexpressed in breast tumors, and EGFR has been suggested to be a therapeutic target in triple-negative (Estrogen Receptor-, Progesterone Receptor-, and HER2-negative) breast cancers [4].

HER2 overexpression in breast cancer is associated with poor prognosis, but the production of HER2-targeted antibodies such as Trastuzumab (Herceptin) and Pertuzumab, and HER2/EGFR tyrosine kinase inhibitors such as Lapatinib, has revolutionized the treatment of HER2-positive breast cancer. Unfortunately, 66–88% of HER2-positive tumors exhibit primary resistance to Trastuzumab as a monotherapy [5–7]. Further, standard Trastuzumab-centered regimens include either a Taxane or an Anthracycline to provide acceptable anti-cancer efficacy, but 15% of patients acquire resistance to these combination therapies as well [8]. These regimens are associated with significant side effects including cardiotoxicity and anaphylaxis [9]. Clearly, additional therapies are needed to reduce the toxicity of these combination therapies and to overcome drug resistance.

A large number of resistance mechanisms to Trastuzumab and Lapatinib have been described (reviewed in [10–13]). Many of these mechanisms involve the ability of these three proteins to function in a partially redundant manner. For example when Trastuzumab inactivates HER2, EGFR and HER3 can still heterodimerize and drive mitogenic and survival signaling [14]. Likewise, Pertuzumab blocks HER2 dimerization with EGFR or HER3, but this does not preclude EGFR/HER3 dimerization and signaling. Lapatinib blocks the kinase activity of both HER2 and EGFR, but while HER3 has very little intrinsic tyrosine kinase activity [15, 16], it can serve as a substrate for c-Met and activate PI3K-dependent signaling in the absence of EGFR and HER2 function [17–19].

Therefore, an improved agent for the treatment of HER2-dependent breast cancer would inactivate EGFR, HER2, and HER3 in parallel, be effective in the treatment of cancer as a single agent, and be mechanistically complementary with the HER2-targeted monoclonal antibodies and tyrosine kinase inhibitors. Herein we describe the identification of a series of molecules that fulfill these criteria.

## RESULTS

### Extracellular EGFR, HER2, and HER3 disulfide bonds as a potential drug target

The anticancer effects of the Disulfide bond Disrupting Agents (DDAs) were discovered serendipitously. Our initial goal was to employ molecular docking and a homology model of the CUB Domain-Containing Protein 1 (CDCP1) to identify compounds that would modulate CDCP1 tyrosine phosphorylation. During this screening, we noticed that compound NSC624205 killed the MDA-MB-468 cells used in the screen when administered at 20 μM for 24 h, while none of the other structurally unrelated compounds tested had this effect. Another cell line used in the initial screens, the BxPC3 pancreatic cell line, was unaffected by the same treatment. Therefore we were intrigued by the selective actions of NSC624205 against MDA-MB-468 cells, but not BxPC3 cells, and endeavored to identify the underlying molecular basis. NSC624205 (structure shown in Figure 4C) contains a sulfinic acid moiety, and the sulfur atom in sulfinic acids can act as a nucleophile with the potential to break disulfide bonds.

**Figure 1:**
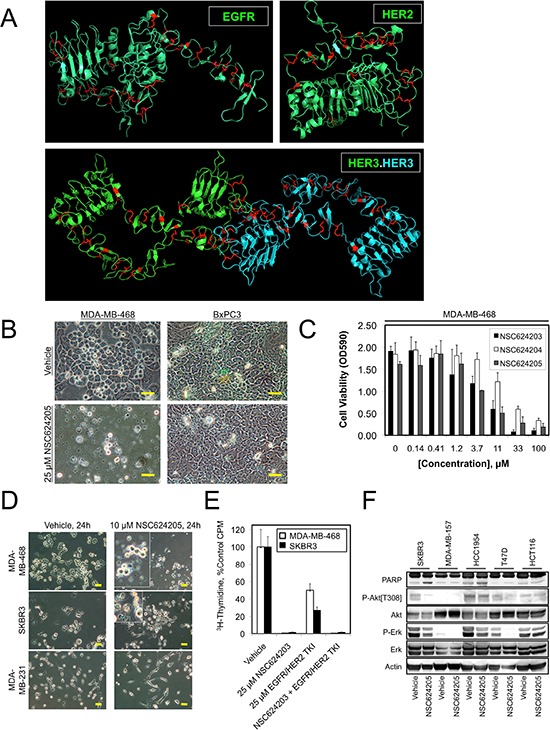
EGFR, HER2, and HER3 Disulfide Bonds as a Therapeutic Target **A.** X-ray crystal structures of the extracellular domains of EGFR, HER2, and HER3 with cysteine residues shown in red. Note the large number of disulfide bonds. **B.** Photomicrographs of MDA-MB-468 or BxPC3 cells treated for 24 hours with 25 μM NSC624205 or the vehicle control. **C.** MDA-MB-468 cells were treated for 24 hours with the indicated concentrations of NSC624203, NSC624204, and NSC624205 and cell viability (cell mass) was measured by crystal violet staining. **D.** Photomicrographs of MDA-MB-468, SKBR3, or MDA-MB-231 cells treated for 24 hours with 10 μM NSC624205 or the vehicle control. **E.** Cell proliferation as measured by thymidine incorporation by MDA-MB-468 and SKBR3 cells treated for 24 hours with NSC624203, an EGFR/HER2 inhibitor, or a combination of the two compounds. **F.** The indicated cancer cell lines were treated for 24 hours with 20 μM NSC624205 or vehicle and cell extracts were analyzed by immunoblot. Actin serves as a loading control. Results in (C) and (E) are presented as the average of triplicate determinations ± S.D. Scale bars are 20 μm.

EGFR, HER2, and HER3 share evolutionarily conserved extracellular domains stabilized by disulfide bonds (Figure 1A) [20–23]. The HER family of receptors is unique among receptor tyrosine kinases in having four extracellular domains, two of which, domains II and IV are cysteine rich (reviewed in [24]). The Insulin receptor subfamily and the Ret tyrosine kinases each contain a single cysteine rich repeat, while none of the remaining 16 subfamilies of receptor tyrosine kinases have a cysteine rich domain.

Structural studies indicate a key role for the cysteine rich repeats in the folding and activation of HER-family receptors. In particular, the ligand-induced conformational change of the extracellular domain of EGFR has been studied extensively (reviewed in [25]). In the structure of the ligand-free EGFR extracellular domain, cysteine rich domain II forms contacts with cysteine rich domain IV to maintain the receptor in the “tethered”, inactive conformation. In the structure of the EGF (or TGFα) ligand-bound, un-tethered, active conformation of the EGFR extracellular domain, a homodimeric structure is formed in which all of the contacts that maintain the dimer involve the cysteine rich domain II of each monomer. Thus, both the inactive and active conformations are maintained by contacts involving cysteine rich domains II and IV or domain II alone, respectively.

According to the data tabulated on the UniProt website (http://www.uniprotorg/uniprot/P00533) EGFR contains 25 intrachain disulfide bonds spanning distances of from four to 30 amino acids in the primary sequence. The loops generated by six pairs of these disulfide bonds are overlapping (e.g. loops corresponding to amino acids 190–199, 194–207; 215–223, 219–231; 232–240, 236–248; 506–515, 510–523; 558–571, 562–579; 620–628, 624–636). Given the intricate and extensive network of disulfide bonding in these receptors that is critical for their proper folding and function, we hypothesized that compounds able to disrupt disulfide bonds might preferentially inactivate HER-family receptor tyrosine kinases and that this might explain the pattern of cancer cell line responsiveness to these agents.

### Sulfinate-containing compounds kill breast cancer cells that overexpress EGFR or HER2 and block Akt phosphorylation

We obtained several sulfinate-containing compounds from the National Cancer Institute's Developmental Therapeutics Program (NCI/DTP) and in a second screen examined their ability to decrease the viability of various human cancer cell lines. As expected, NSC624205 was lethal to MDA-MB-468 breast cancer cells, but had little effect on BxPC3 pancreatic cancer cells, indicating that NSC624205 is not a general cytotoxic agent (Figure 1B). NSC624205 and two related compounds, NSC624203 and NSC624204, decreased cell viability by 50% in the range of 3.7–33 μM (Figure 1C). Over a period of 24 hr, 10 μM NSC624205 killed MDA-MB-468 and SKBR3 cells, which overexpress EGFR and HER2 respectively, but had little effect on the basal-like/triple-negative MDA-MB-231 breast cancer cell line, which does not overexpress either EGFR or HER2 (Figure 1D). Comparison of the ability of NSC624203 to inhibit the proliferation of MDA-MB-468 cells with that of a commercial EGFR/HER2 tyrosine kinase inhibitor (Calbiochem, Cat. # 324673) revealed that NSC624203 more effectively suppressed the proliferation of both cell lines when used at the same concentration (Figure 1E). Examination of the effects of NSC624205 on cell signaling in a small panel of human cancer cell lines demonstrated variable effects depending on the cell line, inhibiting Akt phosphorylation in SKBR3 cells, and Erk phosphorylation in SKBR3, HCC1954, and T47D cells (Figure 1F). Overall, cell killing by the sulfinate compounds correlated most closely with loss of Akt phosphorylation on Thr^308^.

### Sulfinate compounds induce cell death that correlates with EGFR dephosphorylation, PARP cleavage, and reduced ligand-dependent EGFR tyrosine phosphorylation

**Figure 2:**
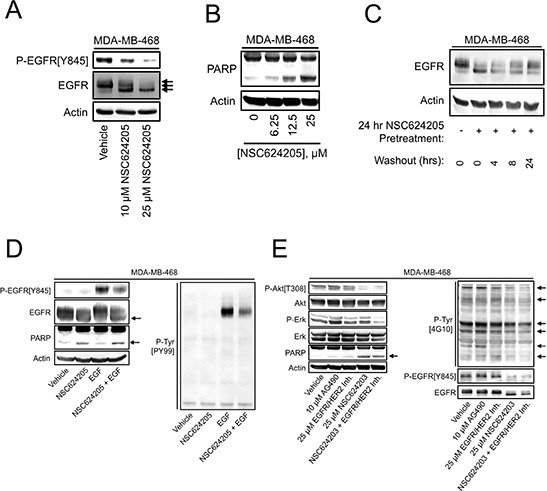
EGFR/HER2/HER3-targeted Compounds Induce Cell Death and Suppress Response to EGF **A.** MDA-MB-468 cells treated as indicated for 24 hours were analyzed by immunoblot for levels of EGFR and EGFR phosphorylation. **B.** MDA-MB-468 cells treated as indicated for 24 hours were analyzed by immunoblot for PARP cleavage. **C.** MDA-MB-468 cells were either left untreated, or treated with 20 μM NSC624205 for 24 hours. NSC624205-treated cells were then washed and incubated for the indicated periods in the absence of drug. EGFR electrophoretic mobility was analyzed by immunoblot. **D.** MDA-MB-468 cells were pretreated with 25 μM NSC624205 or vehicle for 15 hours and then either left untreated or stimulated for 15 minutes with 20 ng/ml EGF, after which cell extracts were analyzed by immunoblot. **E.** MDA-MB-468 cells were treated as indicated for 24 hours and analyzed by immunoblot.

As mentioned above, we initially hypothesized that sulfinate compounds may be useful in destabilizing EGFR-family members; therefore, we examined the effects of NSC624205 on the levels and phosphorylation of EGFR in MDA-MB-468 cells. NSC624205 induced a concentration-dependent increase in EGFR electrophoretic mobility that correlated with a decrease in EGFR phosphorylation detected using a phospho-specific antibody (Figure 2A). NSC624205 also caused a concentration-dependent increase in PARP cleavage, consistent with the induction of apoptosis (Figure 2B). To examine the reversibility of NSC624205 actions, MDA-MB-468 cells were treated for 24 hours with NSC624205 and then the compound was washed out and the cells were allowed to recover for various periods of time. This experiment revealed that at 24 hours post-treatment, EGFR electrophoretic mobility was restored to near control levels, indicating that the effects of this compound are slowly reversible (Figure 2C). To examine whether NSC624205 can suppress cellular responses to EGF, cells were stimulated with EGF in the presence or absence of NSC624205. NSC624205 decreased both overall EGF-induced cellular tyrosine phosphorylation and EGFR tyrosine phosphorylation on Tyr^845^ (Figure 2D). Comparison of NSC624203 with AG490 or an EGFR/HER2 kinase inhibitor (Calbiochem #324673) showed that NSC624203 was more effective at decreasing Akt phosphorylation, increasing PARP cleavage and reducing EGFR tyrosine phosphorylation and overall levels of cellular tyrosine phosphorylation (Figure 2E). Interestingly, combining NSC624203 with the EGFR/HER2 inhibitor blocked Erk phosphorylation more effectively than either drug alone.

### Overexpression of EGFR is sufficient to render breast cancer cells responsive to sulfinate compound-induced toxicity

**Figure 3:**
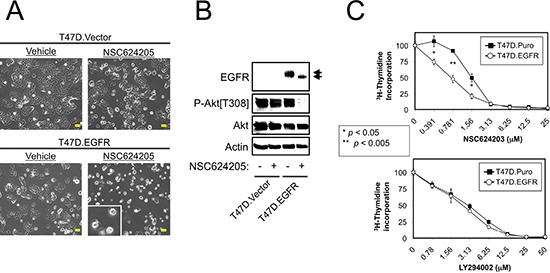
Forced EGFR Expression Sensitizes Cells to EGFR/HER2/HER3-targeted Compounds **A.** Vector control or EGFR overexpressing T47D cells were treated with 20 μM NSC624205 or vehicle for 24 hours and photographed. Extensive cell death was observed in the T47D.EGFR cells, but not the T47D.Vector cells. **B.** Cells treated as in a. were subjected to immunoblot analysis. **C.** Thymidine incorporation of vector control (T47D.Puro) or EGFR overexpressing (T47D.EGFR) cells treated for 24 hours with increasing concentrations of NSC624203 or LY294002. *p* values were calculated using Student's unpaired *t*-test. Results are presented as the average of triplicate determinations ± S.D. Scale bars are 20 μm.

T47D cells are not killed by NSC624205, therefore we examined whether this is because these cells do not overexpress EGFR. Interestingly, T47D cells with enforced EGFR expression underwent cell death in response to NSC624205, but vector control cells did not (Figure 3A). As observed above, NSC624205-mediated cell death correlated with an EGFR electrophoretic mobility shift, and decreased Akt phosphorylation (Figure 3B). Cell proliferation assays showed that similarly, EGFR overexpressing T47D cells were more sensitive to NSC624203 than the control cells (Figure 3C). However, no difference between the two cell lines was observed when proliferation was suppressed using PI3-kinase inhibitor LY294002.

### Structure/activity relationships among sulfinate-containing compounds

**Figure 4:**
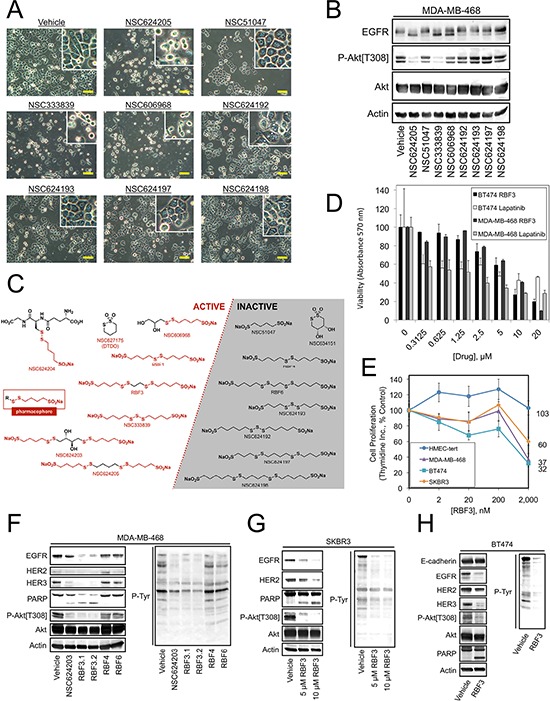
Identification of the Pharmacophore in EGFR/HER2/HER3-targeted Compounds **A.** Photomicrographs of MDA-MB-468 cells treated for 24 hours with 20 μM of the indicated compounds. **B.** Immunoblot analysis of MDA-MB-468 cells treated as in (A) **C.** Chemical structures of Disulfide bond Disrupting Agents (DDAs) showing active compounds on the left side with the pharmacophore highlighted in red, along with the generic pharmacophore. Inactive compounds either lack the pharmacophore sulfinate or disulfide groups, or do not have the appropriate four-carbon spacer between these groups. The exception to this rule is NSC627175/DTDO, which represents a cyclic version of the pharmacophore. **D.** Viability of BT474 or MDA-MB-468 cells treated for 24 hours with the indicated drug at the specified concentrations was measured in MTT assays. **E.** Proliferation of tert-immortalized human mammary epithelial cells (HMEC-tert) and MDA-MB-468, BT474, and SKBR3 breast cancer cells after incubation with the indicated concentrations of RBF3 for 24 hours was measured in thymidine incorporation assays as described in Figure 1E. **F–H.** The indicated cell lines were treated with the specified compounds at 20 μM unless otherwise indicated for 24 hours and analyzed by immunoblot. Assays in (D) and (E) were carried out in triplicate and results were presented as the average ± S.D. Scale bars are 20 μm.

We next screened a panel of sulfinate-containing compounds that are structurally similar to NSC624203 and NSC624205 in order to identify more effective compounds. Analysis of the effects of additional NSC compounds on cell viability (Figure 4A) or EGFR and Akt phosphorylation (Figure 4B) demonstrated that NSC333839 has activity similar to NSC624205. NSC606968 had a partial effect on EGFR electrophoretic mobility, but only a weak effect on Akt phosphorylation. An overall evaluation of these results indicated that all active compounds possess a sulfinate group separated from a disulfide bond by four carbons (Figure 4C, left, boxed). Additional compounds, termed the RBF series, were synthesized to determine whether compounds could be produced that had enhanced activity over the NSC compounds examined initially and to determine whether the sulfinate moiety is required for compound activity. The synthesis of RBF1 was achieved with some modifications of the previously described procedure of Boldyrev and Luzhetskaya [26]. The syntheses of DTDO, RBF2, and RBF3 were previously described by Harpp, et al. [27] and Field and colleagues [28, 29] and could be easily reproduced with minor modifications. Barbee and Field [30] reported the oxidation of a disulfinate to a disulfonate species using H_2_O_2_. Optimization of the reaction time using this oxidant allowed the successful preparation of the disulfonates RBF4 and RBF6 from RBF1 and RBF3, respectively. Oxidation of RBF2 to RBF5 led to the desired compound (as observed by HRMS), however the pure compound could not be separated from other by-products. A summary of the compounds tested and their activity against cancer cells is presented in Figure 4C. Of the RBF series, RBF3 was the most effective and produced a 50% decrease in the viability of MDA-MB-468 cells and the HER2 overexpressing BT474 cells between 5–10 μM (Figure 4D). RBF3 effects on cell proliferation were observed as low as 2 μM, a concentration at which RBF3 had no effect on the proliferation of immortalized human mammary epithelial cells (Figure 4E).

Examination of the biochemical effects of RBF3 on MDA-MB-468 (Figure 4F), SKBR3 (Figure 4G), and BT474 (Figure 4H) cells revealed that RBF3 decreased the levels of EGFR, HER2, and HER3 in parallel. Two independent preparations of RBF3 (i.e., RBF3.1 and RBF3.2) were more effective than NSC624203 at downregulating EGFR and upregulating PARP cleavage in side-by-side comparisons in MDA-MB-468 cells. Therefore, RBF3 was considered to be the most promising lead compound. In contrast to the high activity of RBF1 and RBF3, derivatives of these compounds in which the sulfinate groups had been oxidized to sulfonate groups, RBF4 and RBF6, did not downregulate EGFR, HER2, or HER3 in MDA-MB-468 cells, did not increase PARP cleavage, and did not reduce Akt phosphorylation (Figure 4F). These observations indicate that the sulfinate groups present in these compounds are essential for their activity against cancer cells. Our interpretation of these data is that oxidation of the sulfinate groups to sulfonate causes loss of activity because unlike the sulfinate sulfur, the sulfonate sulfur does not behave as a nucleophile and therefore cannot disrupt the extracellular disulfide bonds in EGFR, HER2, and HER3 and destabilize these proteins.

### Proposed mechanism of action of sulfinate compounds as disulfide-bond disrupting agents (DDAs)

**Figure 5:**
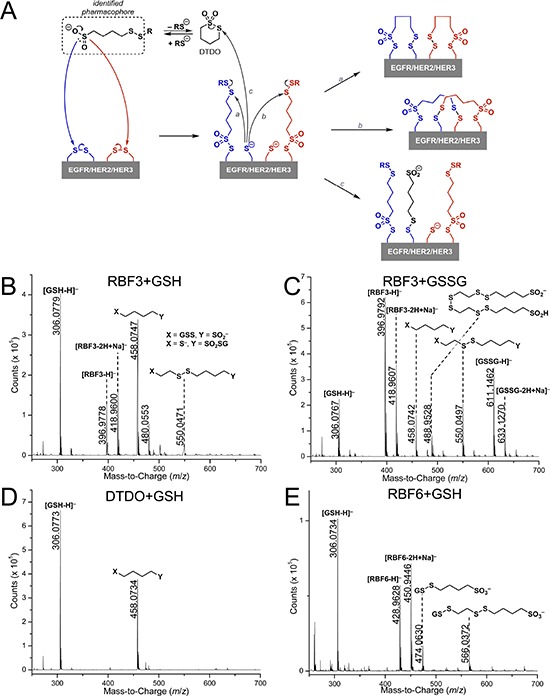
Evidence that EGFR/HER2/HER3-targeted Compounds Can Function as Disulfide Bond Disrupting Agents (DDAs) **A.** Proposed model for how DDAs disrupt disulfide bonds by inserting into them and potentially changing their connectivity *(a* and *b)*. Should cyclization of the pharmacophore occur to form DTDO, reaction with free thiols or thiolates *(c)* may lead to incorporation of the pharmacophore into the protein. **B, C.** Mass spectra of reactions carried out between 10 mM RBF3 and 10 mM reduced (B) or oxidized (C) glutathione for 24 hours at 37°C, pH 7.0. **D, E.** Mass spectra of reactions carried out between 10 mM DTDO (D) or 10 mM RBF6 (E) and 10 mM reduced glutathione for 24 hours at 37°C, pH 7.0. Reactions between DTDO or RBF6 and oxidized glutathione did not lead to products and the related mass spectra are not shown (see SI).

We hypothesize that RBF3 and the other pharmacophore-containing compounds function through the mechanism outlined in Figure 5A, in which the sulfinate sulfur atom mounts a nucleophilic attack on a sulfur atom in a disulfide bond. This would result in disruption of the disulfide bond, the formation of a thiosulfonate group connecting the disulfide bond disrupting agent (DDA) to a Cysteine side chain, and would leave a free thiol group. In a second reaction (pathways *a* and *b*), the disulfide bond within the pharmacophore could react with a free thiol on the protein. Either scenario will result in the insertion of the DDA into a disulfide bond and alteration of the disulfide bond connectivity in the protein, perhaps altering the secondary structure of the protein. A final mechanistic possibility invokes DTDO, a cyclic intermediate accessible from DDAs with appropriate spacing between the sulfinate and disulfide functions (vide infra). Reaction between DTDO and a free thiol on the protein (pathway *c*) could then disrupt the protein disulfide infrastructure through (potentially transient) DTDO incorporation.

In support of this mechanistic hypothesis, electrospray ionization (ESI) mass spectrometric analysis of reactions between RBF3, RBF6, or DTDO and glutathione in both the oxidized (GSSG) and reduced form (GSH) were performed in order to identify products from the reaction of DDAs with a biological thiol or disulfide (Figures 5B–E). Reaction of RBF3 with GSH led to two product masses (*m*/*z* = 458 and *m*/*z* = 550) from incorporation of structural segments of RBF3 to glutathione (Figure 5B). While the precise structure (i.e., with respect to X and Y in the figure) cannot be assigned by ESI-MS, the result suggests that free thiols can react with the disulfide bonds within the RBF3 structure. Although decomposition of RBF3 to DTDO is possible [29], the formation of a product featuring incorporation of both the pharmacophore and the two carbon linker (*m*/*z* = 550, Figure 5B) suggests that the free thiols present in solution can directly attack the disulfide bonds of RBF3. Reaction of RBF3 with GSSG (Figure 5C) led to the same ensemble of products observed for the combination of RBF3 and GSH, indicating cleavage of the disulfide bond in GSSG. In fact, formation of GSH was observed when GSSG was exposed to RBF3 (Figure 5C). This result is consistent with the hypothesis that the nucleophilic sulfinate groups of RBF3 can attack disulfide bonds, releasing a thiolate and incorporating RBF3 to a biomolecule. Formation of an ion with *m*/*z* = 489 was also observed and its exact mass agrees with the structure assigned in Figure 5C. Its formation is possible through the disruption of the disulfide bonds within the RBF3 structure by the sulfinate groups. A control study where a solution of RBF3 itself was exposed to the same conditions led to the identification of the same ion, indicating that this species is not produced from reactions between RBF3 and glutathione. DTDO was also studied in such reactions. As expected, reaction of DTDO with GSH led to incorporation of the pharmacophore to glutathione (*m*/*z* = 458, Figure 5D, akin to pathway *c* in Figure 5A) while reaction with GSSG gave no reaction (Supplemental Data, Figure S1). Reaction of RBF6 with GSH (Figure 5E) led to the incorporation of segments from RBF6 to glutathione (*m*/*z* = 474 and 566). On the other hand, reaction of RBF6 with GSSG (Supplemental Data, Figure S2) did not result in the formation of any products. These results are consistent with the hypothesis that the thiol group of GSH may attack the disulfide bonds within the structure of the DDAs and suggests that the nucleophilic sulfinate moiety is required to disrupt the disulfide bonds within a biomolecule.

To summarize, the biochemical functions of RBF3 require two chemical moieties, the sulfinate group, which may function as a nucleophile to break disulfide bonds, and a disulfide bond that is susceptible to nucleophilic attack. Further, this donor/acceptor combination must be separated by four intervening carbon atoms, suggesting that DTDO may function as an intermediate that is capable of functioning as a target of thiolate nucleophilic attack.

### DDAs exhibit anti-cancer activity *in vivo* without evidence of toxicity

**Figure 6:**
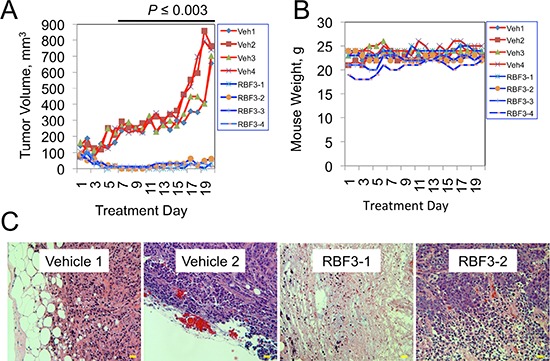
DDAs Suppress Tumor Growth Without Evidence of Toxicity **A.** Growth of tumors derived from BT474 cells in mice treated with either Vehicle (water; red lines) or 40 mg/kg RBF3 (blue lines). Animals were treated by intraperitoneal injections administered once daily, Monday–Friday. **B.** Plot of animal weights over time. **C.** Photomicrographs of hematoxylin and eosin (H&E) stained sections of tumors from vehicle- or RBF3-treated mice. Note the presence of extensive necrosis in the RBF3-treated tumors. Scale bars are 20 μm.

Given the promising negative impact of RBF3 on the viability of HER2 and EGFR overexpressing breast cancer cell lines, we examined whether RBF3 had activity against xenografts of human breast cancer. Strikingly, 40 mg/kg RBF3 strongly suppressed the growth of tumors derived from BT474 cells (Figure 6A). In contrast, in vehicle (water) treated animals the tumors grew rapidly. During the treatment period the weights of the animals were not significantly affected by drug treatment (Figure 6B). Examination of the histology of the remnants of RBF3 treated tumors revealed that most of the tumor tissue was necrotic or fibrotic, and that only a small fraction of these tumors was composed of viable cancer cells (Figure 6C). In separate experiments, we treated tumor-bearing mice with RBF3 at dosages of up to 160 mg/kg/day. Under these conditions, no evidence of toxicity was observed based on histological examination of kidney and liver tissue (Supplemental Data, Figure S1). In contrast, tumor tissues from RBF3-treated animals exhibited a high frequency of cancer cell death.

## DISCUSSION

Conventional drugs that act on HER2, EGFR, and HER3 include monoclonal antibodies or tyrosine kinase inhibitors. DDAs represent a new way of inactivating these oncogenes by protein downregulation. To our knowledge this approach has not previously been taken with receptor tyrosine kinases, but there is precedence for related approaches for other drug targets. The breast cancer drug Fulvestrant functions in part by downregulating the Estrogen Receptor [31]. Further, we previously described a new class of Cyclin-Dependent Kinase (CDK) inhibitors that inhibit cell proliferation by inducing CDK aggregation and degradation via aggresomes [32]. The mechanisms by which DDAs downregulate EGFR family members are currently under investigation, and could involve proteasomal or lysosomal degradation, and aggresomes or autophagosomes, respectively. The conserved disulfide bonding pattern in the extracellular domains of EGFR family members may provide an additional, complementary approach for targeting these oncogenes.

The first DDAs that we investigated, NSC624203 and NSC624205, were originally designed as part of an effort to produce compounds that were protective against radiotoxicity [28, 33]. In these studies, these and similar sulfur-rich compounds were administered to mice at very high dosages before toxicity was observed.

Our results showing that activity against cancer cells requires compounds with a sulfinate group attached to a disulfide bond by four intervening carbon atoms indicate that this pharmacophore has biological activity distinct from radioprotection. The precise spacing required between the disulfide and sulfinate groups, and the fact that a sulfonate group cannot substitute for the sulfinate group, suggest that the DDA pharmacophore may require the formation of a cyclic intermediate for its activity. Alternately, the requirement for precise spacing may result from the distances between the repetitive disulfide bonded structures in the extracellular domains of EGFR, HER2, and HER3 (see Figure 1A).

This class of compounds has previously been shown to react intramolecularly to form DTDO [29]. Thus, although a previous report suggested that RBF3 does not form significant amounts of DTDO in a 16 hour period [29], it is possible that both reductive cleavage of disulfide bonds by the sulfinate group and DTDO formation contribute to the anticancer actions of RBF3. DTDO and related compounds were previously shown to exhibit activity against human immunodeficiency virus type I through chemical modification of the HIV-1 nucleocapsid zinc finger protein NCp7, resulting in ejection of the bound zinc ligand from the protein [34]. DTDO formation might likewise inactivate cellular zinc finger proteins or enzymes with essential cysteine residues at their active sites.

It is clear however that the sulfinate group is essential for the downregulation of EGFR, HER2, and HER3 because DTDO does not downregulate these proteins in cell culture. Further, DTDO can react with a free thiol (e.g. GSH, Figure 5D), but is unable to react with disulfide bonds *in vitro* (e.g. GSSG, Supplemental Data Figure S2). The importance of DTDO formation in the anticancer actions of RBF3 is currently being investigated.

It is possible that one of the reasons that RBF3 and similar compounds have little observable toxicity is due to the two charged sulfinic acid groups. This may render these molecules poorly membrane permeable and restrict their effects primarily to extracellular proteins and the extracellular domains of transmembrane proteins, without altering the activity of intracellular proteins. Further work is required to optimize these agents with respect to their absorption and stability *in vivo*.

In summary, DDAs show impressive anticancer activity without obvious toxicity in mice. This class of agents might be useful in the treatment of HER2- and EGFR-dependent breast tumors and may be effective for the treatment of cancers that have acquired resistance to monoclonal antibodies or tyrosine kinase inhibitors targeting these enzymes. The DDA pharmacophore may also find more widespread use in drug development beyond the treatment of HER2- and EGFR-driven human cancers. The presence of two sulfur–containing moieties within the pharmacophore structure generates a combined transiency of their oxidation states (sulfinate to thiosulfonate and disulfide to thiol) that makes these molecules interesting and unique. This intramolecular chemical diversity may have broad applications in medicinal chemistry.

## MATERIALS AND METHODS

### Compound synthesis, purification, and analysis

#### General methods

Reagents and solvents were purchased from commercial sources and used without further purification unless otherwise specified. ^1^H and ^13^C NMR spectra were recorded using commercially-obtained (Cambridge Isotope Laboratories) deuterated solvents on Varian Inova-500 (^1^H at 500 MHz; ^13^C at 125 MHz) or Mercury-300 (^1^H at 300 MHz; ^13^C at 75 MHz) spectrometers. Chemical shifts (δ) are given in parts per million (ppm) relative to tetramethylsilane (TMS) and referenced to residual protonated solvent (CDCl_3_: δ H 7.26 ppm, δ C 77.23 ppm; D_2_O: δ H 4.79 ppm). Coupling constants are given in Hz. Spin multiplicities are presented by the following symbols: s (singlet), bs (broad singlet), d (doublet), t (triplet), q (quartet), p (pentet), and m (multiplet). Electrospray ionization (ESI) high resolution mass spectra (HRMS) were recorded on an Agilent 6200 ESI-TOF instrument operating in positive or negative ion mode as stated, with methanol as the carrier solvent. NMR spectra of synthesized compounds are shown in Figure S3.

#### 1, 2-Dithiane-1, 1-dioxide (DTDO)

DTDO was prepared using a procedure reported by Harpp, et al. with some modifications [27]. A solution of 1,4-butanedithiol (24.7 mmol, 2.90 mL) in AcOH (25 mL) was cooled in an ice bath and a 30% aqueous H_2_O_2_ solution (8.8 mL) was added slowly such that the temperature did not rise above 35°C. After stirring for 18 h, the solvent was removed under vacuum, and the residue was diluted with water (25 mL), neutralized with NaHCO_3_, and extracted with toluene (4 × 50 mL). The organic extract was dried with MgSO_4_, and the solvent was removed under vacuum. The resulting solid was recrystallized three times from Et_2_O to afford 1.55 g (10.2 mmol, 41% yield) of the product as a white solid. mp 55–58°C (lit. [27] mp 54–56°C); ^1^H NMR (CDCl_3_, 500 MHz): δ2.03 (2H, m), 2.37 (2H, m), 3.35 (4H, m) ppm; ^13^C NMR (CDCl_3_, 125 Hz): δ 25.1, 26.2, 35.2, 59.7 ppm.

#### Sodium 4-({4-[(sodiooxy)sulfinyl]butyl}disulfanyl)butane-1-sulfinate (RBF1)

RBF1 was prepared using a modified version of the procedure originally described by Boldyrev and Luzhetskaya [26]. To a solution of DTDO (0.389 g, 2.56 mmol) in anhydrous MeOH (6.4 mL) at room temperature (rt) under argon atmosphere, a solution of NaOMe (prepared from 58.9 mg of Na^0^ in 5.1 mL of anhydrous MeOH) was added dropwise. The mixture was kept stirring for 5 h. After this period, the reaction mixture was concentrated under vacuum until a precipitate was formed and acetone was added to complete the precipitation. The solid was filtered, washed with acetone (3 × 10 mL), and dried under reduced pressure to afford 0.272 g (0.775 mmol, 61% yield) of the product as a white solid. ^1^H NMR (D_2_O, 300 MHz): δ 1.58–1.90 (8H, m), 2.37 (4H, t, *J* = 7.5 Hz), 2.77 (4H, t, *J* = 6.0 Hz) ppm; ^13^C NMR (D_2_O, 75 MHz): δ 20.7, 27.9, 37.7, 60.3 ppm; HRMS-ESI: *m/z* [M–Na]^−^ calcd for [C_8_H_16_NaO_4_S_4_]^−^: 326.9835; found: 326.9828.

#### Sodium 4-[({4-[(sodiooxy)sulfinyl]butyl}sulfanyl)disulfanyl]butane-1-sulfinate (RBF2)

RBF2 was prepared according to the procedure described by Field and Khim [28] to afford 0.405 g (1.06 mmol, 78% yield) of the desired product as a white solid. ^1^H NMR (D_2_O, 500 MHz): δ 1.72 (4H, p, *J* = 7.5 Hz), 1.90 (4H, p, *J* = 7.5 Hz), 2.41 (4H, t, *J* = 7.5 Hz), 2.99 (4H, t, *J* = 7.5 Hz) ppm; ^13^C NMR (D_2_O, 125 MHz): δ 20.6, 27.5, 37.8, 60.2 ppm; HRMS-ESI: *m/z* [M–Na]^−^ calcd for [C_8_H_16_NaO_4_S_5_]^−^: 358.95553; found: 358.95594.

#### Sodium 4-{[2-({4-[(sodiooxy)sulfinyl]butyl}disulfanyl)ethyl]disulfanyl}butane-1-sulfinate (RBF3)

RBF3 was prepared according to the procedure described by Srivastava and Field [29] to afford 0.315 g (0.711 mmol, 65% yield) of the desired product as a white solid. ^1^H NMR (D_2_O, 500 MHz): δ 1.71 (4H, p, *J* = 7.5 Hz), 1.86 (4H, p, *J* = 7.5 Hz), 2.41 (4H, t, *J* = 7.5 Hz), 2.84 (4H, t, *J* = 6.0 Hz), 3.12 (4H, s) ppm; ^13^C NMR (D_2_O, 125 MHz): δ 20.8, 28.0, 37.0, 37.8, 60.4 ppm; HRMS-ESI: *m/z* [M+H]^+^ calcd for [C_10_H_21_Na_2_O_4_S_6_]^+^: 442.9554; found: 442.9554. The ^1^H NMR data is consistent with the literature [29].

#### Optimization of reaction time for oxidation of RBF1 to sodium 4-({4-[(sodiooxy)sulfonyl]butyl}disulfanyl)butane-1-sulfonate (RBF4)

To a solution of RBF1 (0.102 g, 0.292 mmol) in DI water (4.3 mL) with stirring at rt, a solution of H_2_O_2_ (72 μL of a 30% aqueous H_2_O_2_ solution in 1.05 mL of DI water) was added dropwise. After different reaction periods (i.e., immediately after addition, 30 min, and 1, 2, 7, 24, or 48 h), the water was evaporated under vacuum and the resulting solid was analyzed by ^1^H NMR. The absence of peaks related to the starting material and appearance of peaks associated with the desired oxidation product in every reaction period indicated fast conversion. Additional peaks were observed after 2 hours and total degradation of the desired product to an overoxidized species, butane-1, 4-disulfinate, was observed after 48 h. An optimized reaction time of 15 min was selected to avoid both the presence of unreacted starting material and degradation products.

#### Sodium butane-1, 4-disulfinate

^1^H NMR (D_2_O, 500 MHz): δ 1.84 (4H, bs), 2.93 (4H, bs) ppm; ^13^C NMR (D_2_O, 125 MHz): δ 23.0, 50.4 ppm; HRMS-ESI: *m/z* [M–Na]^−^ calcd for [C_4_H_8_NaO_6_S_2_]^−^: 238.9665; found: 238.9613. The ^1^H and ^13^C NMR data are consistent with the Spectral Database for Organic Compounds (SDBSWeb: http://sdbs.db.aist.go.jp (National Institute of Advanced Industrial Science and Technology, accessed 9/12/14); SDBS No.: 5345).

#### RBF4

To a solution of RBF1 (78.2 mg, 0.223 mmol) in DI water (3.3 mL) with stirring at rt was added dropwise a solution of H_2_O_2_ (55 μL of a 30% aqueous H_2_O_2_ solution in 0.82 mL of DI water). After 15 min, the water was evaporated under vacuum and the resulting solid was washed with hot solvents (acetone, *i*-PrOH, and EtOH). The solid was collected by vacuum filtration and dried under reduced pressure to afford 33 mg (0.086 mmol, 30% yield) of the product as a white solid. ^1^H NMR (D_2_O, 500 MHz): δ 1.85 (8H, bs), 2.80 (4H, bs), 2.95 (4H, bs) ppm; ^13^C NMR (D_2_O, 125 MHz): δ 23.0, 27.4, 37.5, 50.6 ppm; HRMS-ESI: *m/z* [M–Na]^−^ calcd for [C_8_H_16_NaO_6_S_4_]^−^: 326.9733; found: 358.9739.

#### Sodium 4-{[2-({4-[(sodiooxy)sulfonyl]butyl}disulfanyl)ethyl]disulfanyl}butane-1-sulfonate (RBF6)

To a solution of RBF3 (0.129 g, 0.292 mmol) in DI water (4.3 mL) with stirring at rt was added dropwise a solution of H_2_O_2_ (72 μL of a 30% aqueous H_2_O_2_ solution in 1.05 mL of DI water). After 15 min, the water was evaporated under vacuum and the resulting solid was washed with hot solvents (acetone, *i*-PrOH, and EtOH). The solid was collected by vacuum filtration and dried under reduced pressure to afford 0.134 g (0.282 mmol, 97% yield) of the product as a white solid. ^1^H NMR (D_2_O, 500 MHz): δ 1.89 (8H, bs), 2.86 (4H, bs), 2.98 (4H, bs), 3.13 (4H, bs) ppm; ^13^C NMR (D_2_O, 125 MHz): δ 23.0, 27.4, 37.0, 37.6, 50.6 ppm; HRMS-ESI: *m/z* [M–Na]^−^ calcd for [C_10_H_20_NaO_6_S_6_]^−^: 450.9487; found: 450.9499.

### Cell culture, cell extraction, and immunoblot analysis

Cancer cell lines were purchased from ATCC, Manassas, VA. Human Mammary Epithelial cells immortalized by telomerase expression (*tert*-HMEC) have been described previously [35]. Cells were cultured in Dulbecco's Modified Eagle's Medium supplemented with 10% fetal bovine serum. Cell extracts were prepared as described previously [36] and immunoblot analysis was carried out with the following antibodies: EGFR (#4267), phospho-EGFR[Y845] (#6963), PARP (#9532), phospho-Akt[T308] (#9275), Akt (#4691), phospho-Erk (#9101), HER2 (#2165), HER3 (#4754), phospho-HER2[Y877] (#2241), and phospho-S6 (#2211) from Cell Signaling Technology, Danvers, MA; Erk (sc-93), phospho-Tyrosine (sc-7020), and Actin (sc-1616) from Santa Cruz Biotechnology, Santa Cruz, CA; phospho-Tyrosine (4G-10, 05–321) from Millipore, Temecula, CA; E-cadherin (610182) from BD Transduction Laboratories, San Jose, CA.

### Construction of stable cell lines

T47D cells stably overexpressing EGFR were constructed using the previously described retroviral vector [37] purchased from Addgene (Plasmid #11011), Cambridge, MA. Retrovirus was produced using the amphotropic Phoenix cell line [38] and transduced cultures were selected with 5 μg/ml puromycin.

### Cell proliferation and viability assays

Cell proliferation was measured by thymidine incorporation as described previously [36]. Briefly, unless otherwise stated cells were treated for 24 hours with various compounds and cellular DNA was labeled with ^3^H-thymidine for three hours. Cell viability was assessed using either crystal violet staining or the MTT (3-(4, 5-Dimethyl-2-thiazolyl)-2, 5-diphenyl-2H-tetrazolium bromide) assay. Crystal violet assays were carried out according to an online protocol (http://www.whitelabs.org/) and crystal violet binding to cells was quantified by elution with methanol and measuring absorption at 590 nm. MTT assays were carried out according to the Manufacturer's instructions (kit CGD1, Sigma-Aldrich, St. Louis, MO).

### Software used to visualize protein structure and compound chemical structure

Protein structures were visualized using MacPyMol v0.99 2006, DeLano Scientific LLC, San Carlos, CA. Chemical structures were drawn using ChemBioDraw^®^ Ultra 13.0, CambridgeSoft, PerkinElmer, Waltham, MA.

### *In vivo* tumor studies and histochemical analysis of tumor tissue

All experimental protocols were approved by the Institutional Animal Care and Use Committee of the University of Florida. Tumors were initiated by the injection of 2 × 10^6^ BT474 cells into the mammary fat pads of female NSG mice (005557, The Jackson Laboratory, Bar Harbor, ME) in sterile saline. Mice with palpable tumors were randomly assigned to vehicle (water) or 40 mg/kg RBF3 treatment groups for tumor growth studies. Treatments were administered Monday through Friday by intraperitoneal injection. Tumor volumes were estimated using the formula: Volume = (L*L*W)/2, where tumor length, L > tumor width, W. Tumors and other tissues were fixed with 4% paraformaldehyde in phosphate-buffered saline. Tissues were paraffin embedded and sections were cut and stained with hematoxylin and eosin (H&E) by the University of Florida Molecular Pathology Core (http://molecular.pathology.ufl.edu/).

### Analysis of DDA chemical reactivity by mass spectrometry

#### Incubation of RBF3

A solution of RBF3 (8.9 mg, 2.0 × 10^− 2^ mmol) in 1.98 mL of DI water with 20 μL of 0.10 M phosphate buffer (pH = 7.4) was stirred for 24 hours at 37°C (in an oil bath) in a capped vial. After this period, the solution was cooled to rt and an aliquot was collected and analyzed by MS.

#### Reaction of RBF3 with GSH

To a solution of RBF3 (8.9 mg, 2.0 × 10^− 2^ mmol) in 980 μL of DI water with 20 μL of 0.10 M phosphate buffer (pH = 7.4) at rt, 1.00 mL of 2.0 × 10^− 2^ M GSH in DI water was added. The reaction was stirred for 24 hours at 37°C (in an oil bath) in a capped vial. After the reaction period, the solution was cooled to rt and an aliquot was collected and analyzed by MS.

#### Reaction of RBF3 with GSSG

To a solution of RBF3 (8.9 mg, 2.0 × 10^− 2^ mmol) in 650 μL of DI water with 20 μL of 0.10 M phosphate buffer (pH = 7.4) at rt, 1.33 mL of 1.5 × 10^− 2^ M GSSG in DI water was added. The reaction was stirred for 24 hours at 37°C (in an oil bath) in a capped vial. After the reaction period, the solution was cooled to rt and an aliquot was collected and analyzed by MS.

#### Reaction of DTDO with GSH

To a suspension of DTDO (3.0 mg, 2.0 × 10^− 2^ mmol) in 980 μL of DI water with 20 μL of 0.10 M phosphate buffer (pH = 7.4) at rt, 1.00 mL of 2.0 × 10^− 2^ M GSH in DI water was added. The reaction was stirred for 24 hours at 37°C (in an oil bath) in a capped vial. After the reaction period, the solution was cooled to rt and an aliquot was collected and analyzed by MS.

#### Reaction of DTDO with GSSG

To a suspension of DTDO (3.0 mg, 2.0 × 10^− 2^ mmol) in 650 μL of DI water with 20 μL of 0.10 M phosphate buffer (pH = 7.4) at rt, 1.33 mL of 1.5 × 10^− 2^ M GSSG in DI water was added. The reaction was stirred for 24 hours at 37°C (in an oil bath) in a capped vial. After the reaction period, the solution was cooled to rt and an aliquot was collected and analyzed by MS.

#### Reaction of DTDO with GSH and GSSG

To a suspension of DTDO (3.0 mg, 2.0 × 10^− 2^ mmol) in 815 μL of DI water with 20 μL of 0.10 M phosphate buffer (pH = 7.4) at rt, 670 μL of 1.5 × 10^− 2^ M GSSG and 500 μL of 2.0 × 10^− 2^ M GSH in DI water were added. The reaction was stirred for 24 hours at 37°C (in an oil bath) in a capped vial. After the reaction period, the solution was cooled to rt and an aliquot was collected and analyzed by MS.

#### Incubation of RBF6

A solution of RBF6 (9.5 mg, 2.0 × 10^− 2^ mmol) in 1.98 mL of DI water with 20 μL of 0.10 M phosphate buffer (pH = 7.4) was stirred for 24 hours at 37°C (in an oil bath) in a capped vial. After this period, the solution was cooled to rt and an aliquot was collected and analyzed by MS.

#### Reaction of RBF6 with GSH

To a solution of RBF6 (9.5 mg, 2.0 × 10^− 2^ mmol) in 980 μL of DI water with 20 μL of 0.10 M phosphate buffer (pH = 7.4) at rt, 1.00 mL of 2.0 × 10^− 2^ M GSH in DI water was added. The reaction was stirred for 24 hours at 37°C (in an oil bath) in a capped vial. After the reaction period, the solution was cooled to rt and an aliquot was collected and analyzed by MS.

#### Reaction of RBF6 with GSSG

To a solution of RBF6 (9.5 mg, 2.0 × 10^− 2^ mmol) in 650 μL of DI water with 20 μL of 0.10 M phosphate buffer (pH = 7.4) at rt, 1.33 mL of 1.5 × 10^− 2^ M GSSG in DI water was added. The reaction was stirred for 24 hours at 37°C (in an oil bath) in a capped vial. After the reaction period, the solution was cooled to rt and an aliquot was collected and analyzed by MS.

## SUPPLEMENTARY FIGURES


