# Supplementary material for: Novel agents that downregulate EGFR, HER2, and HER3 in parallel
Source: Oncotarget. 2015 Mar 19;6(12):10445–59. doi: 10.18632/oncotarget.3398 (PMC4496366; doi:10.18632/oncotarget.3398)
Supplement: Supplementary file 1 [file oncotarget-06-10445-s001.pdf]

SUPPLEMENTARY FIGURES

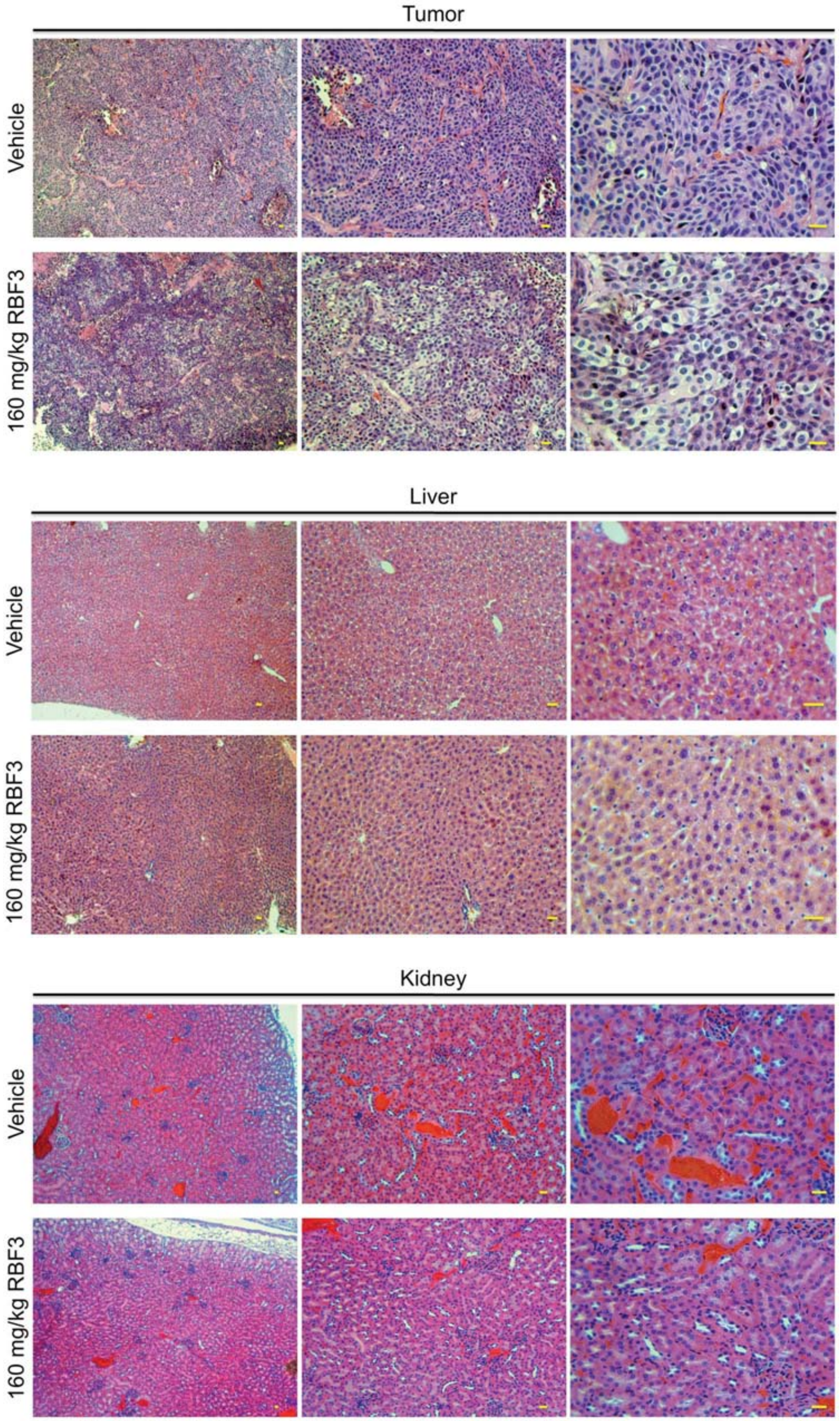

Supplementary Figure S1: RBF3 (160 mg/kg) induces a high frequency of cancer cell death without evidence of liver or kidney toxicity (Scale Bars = 20 µm).

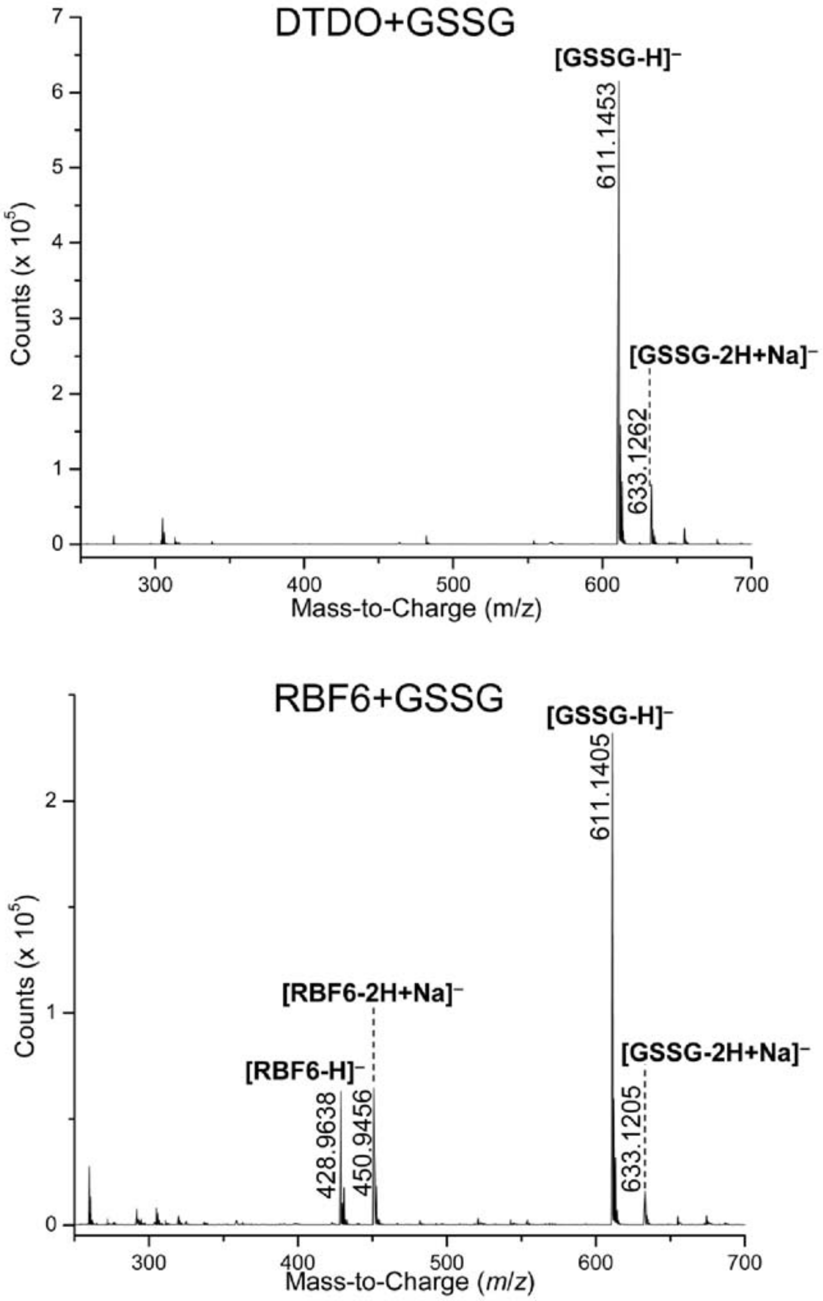

Supplementary Figure S2: Mass spectra of reactions carried out between 10 mM DTDO or 10 mM RBF6 with 10 mM oxidized glutathione (GSSG) for 24 hours at 37°C, pH 7.0.

Supplementary Figure S3: NMR spectra of the synthesized compounds.

DTDO

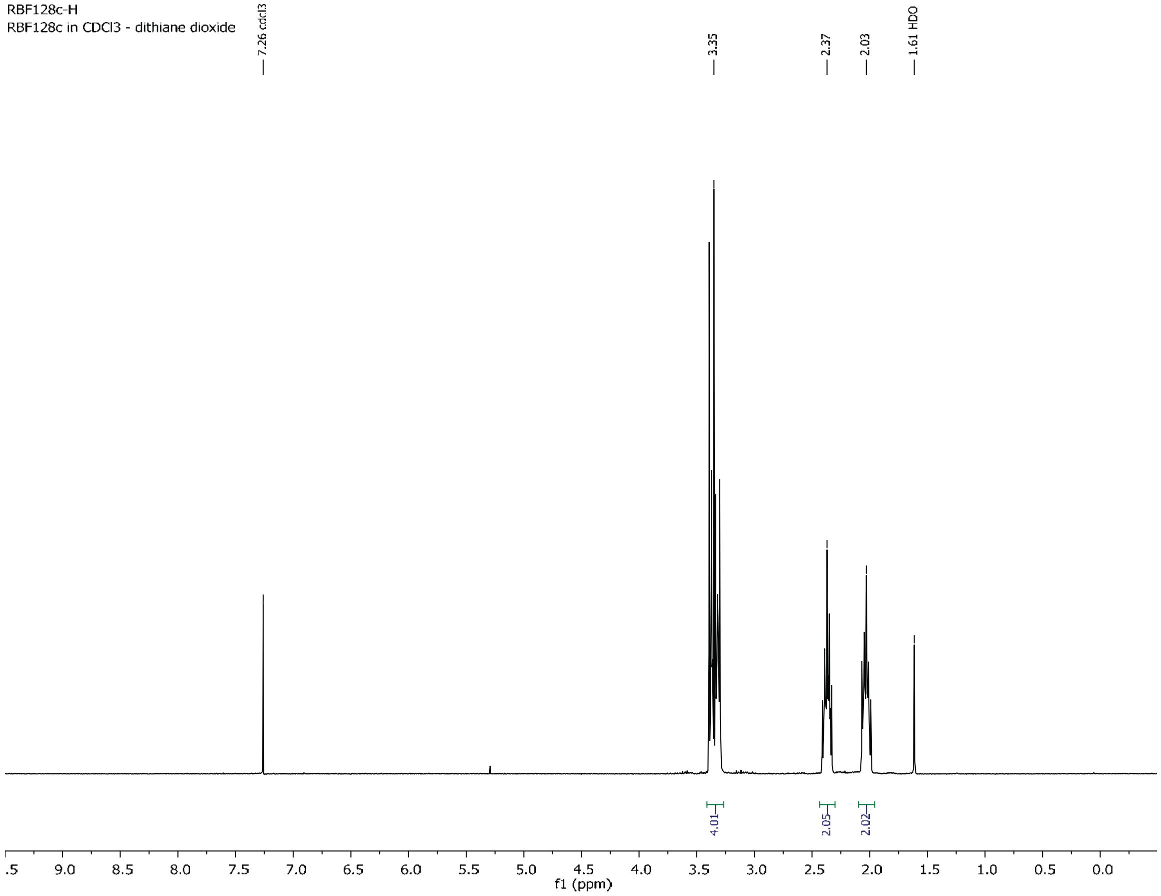

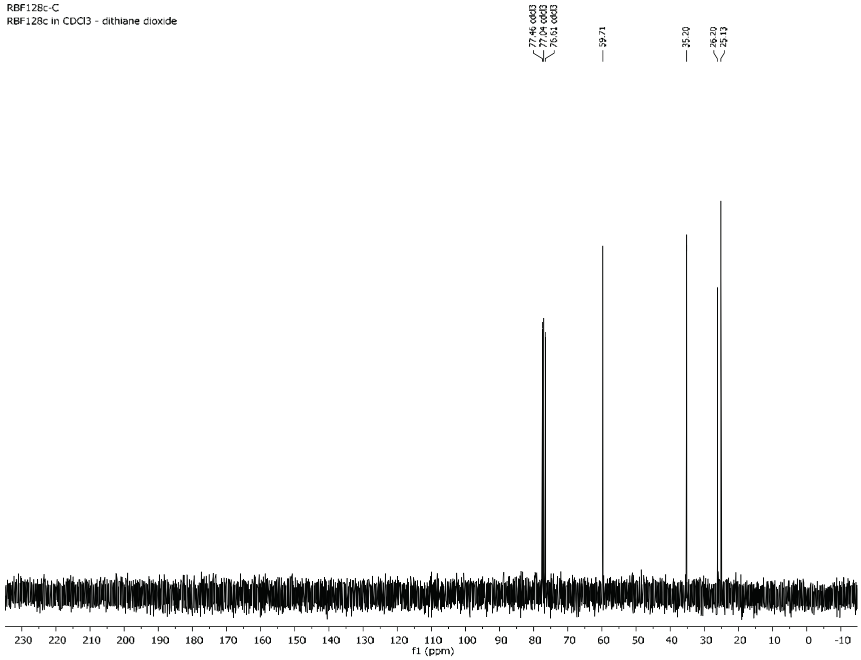

(*Continued*)

RBF1

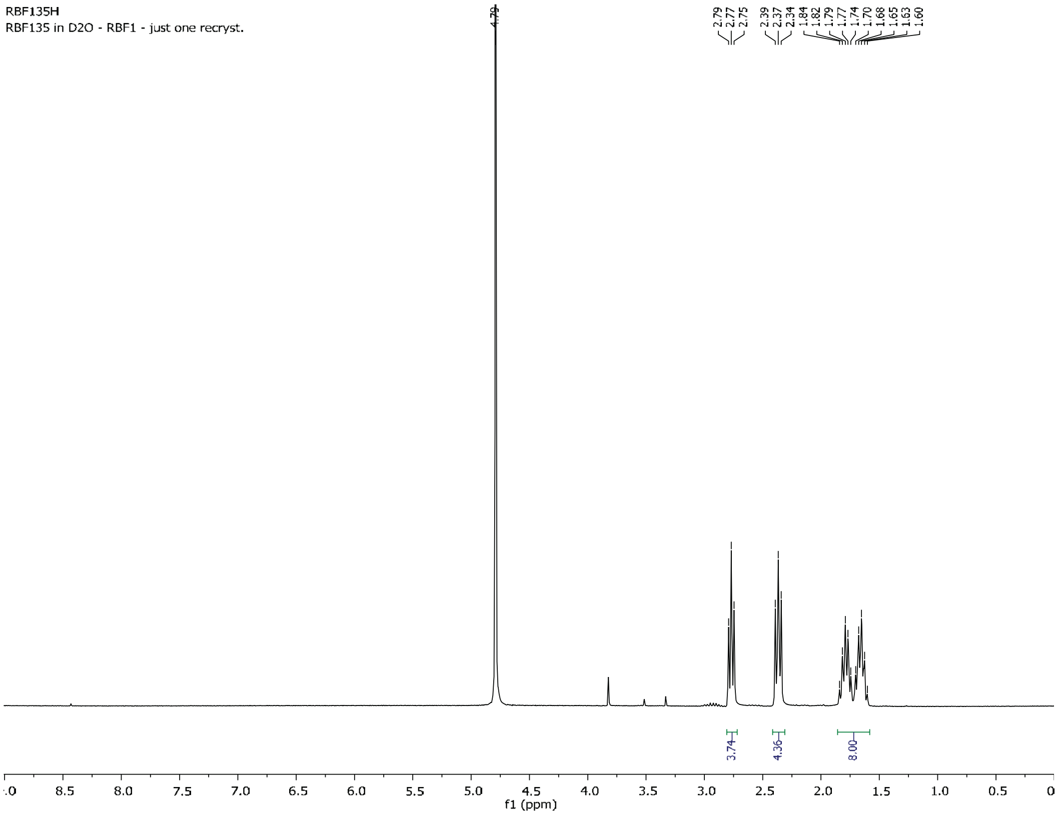

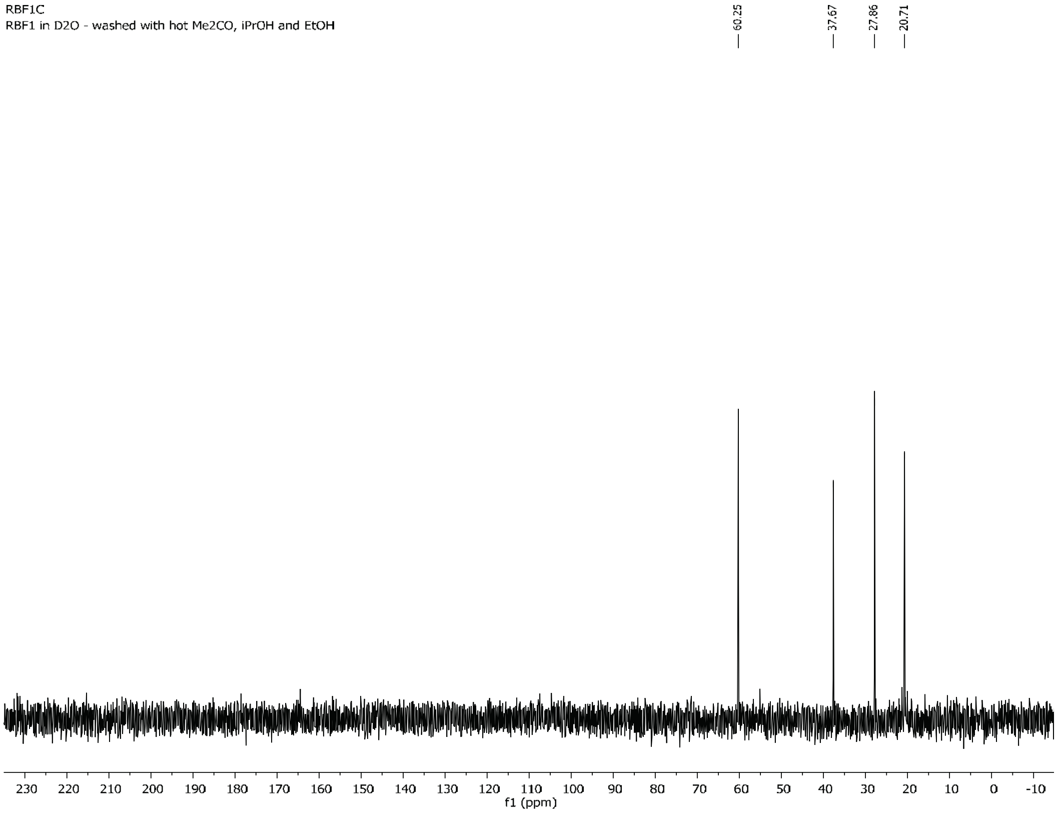

(*Continued*)

RBF2

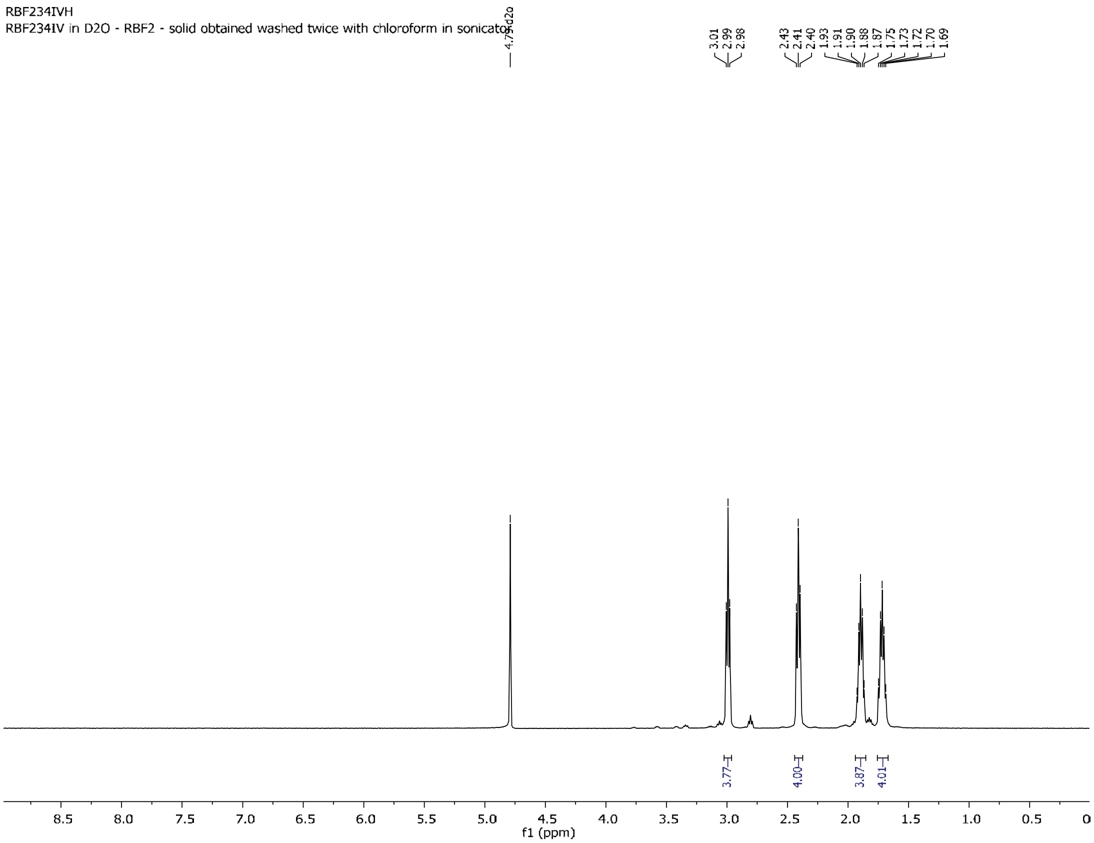

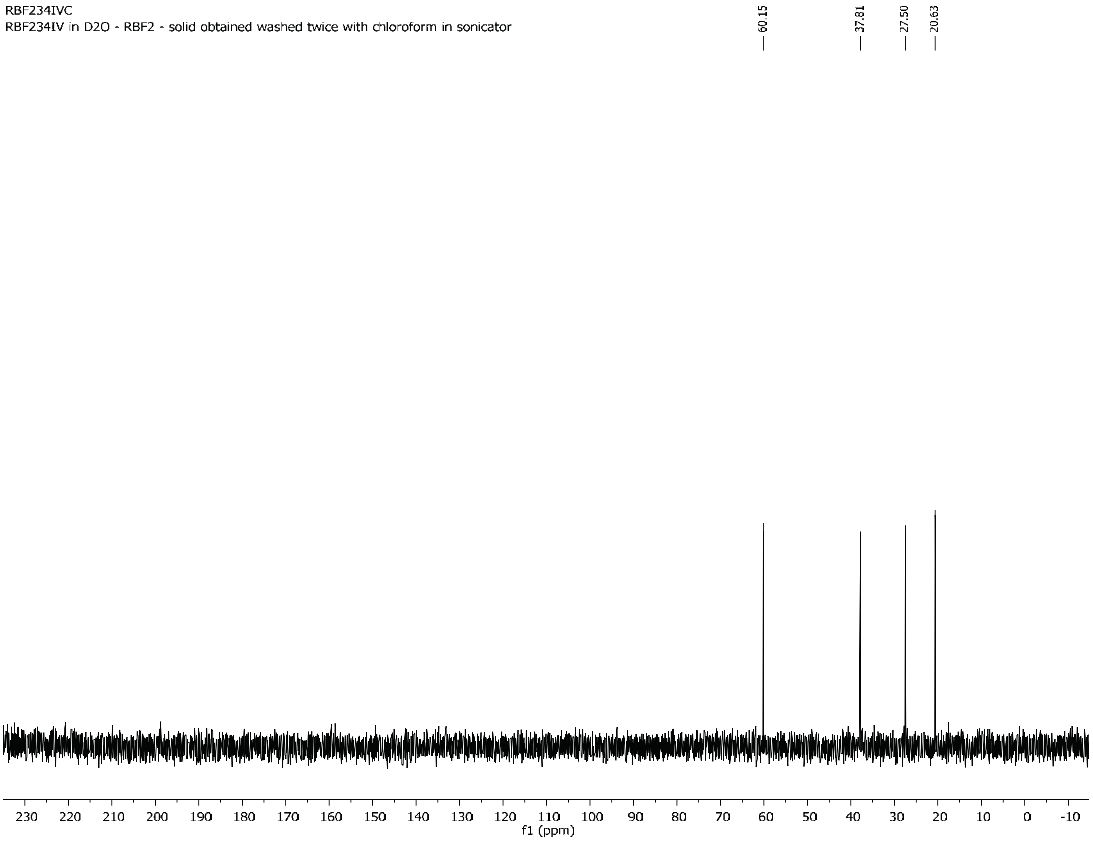

(*Continued*)

RBF3

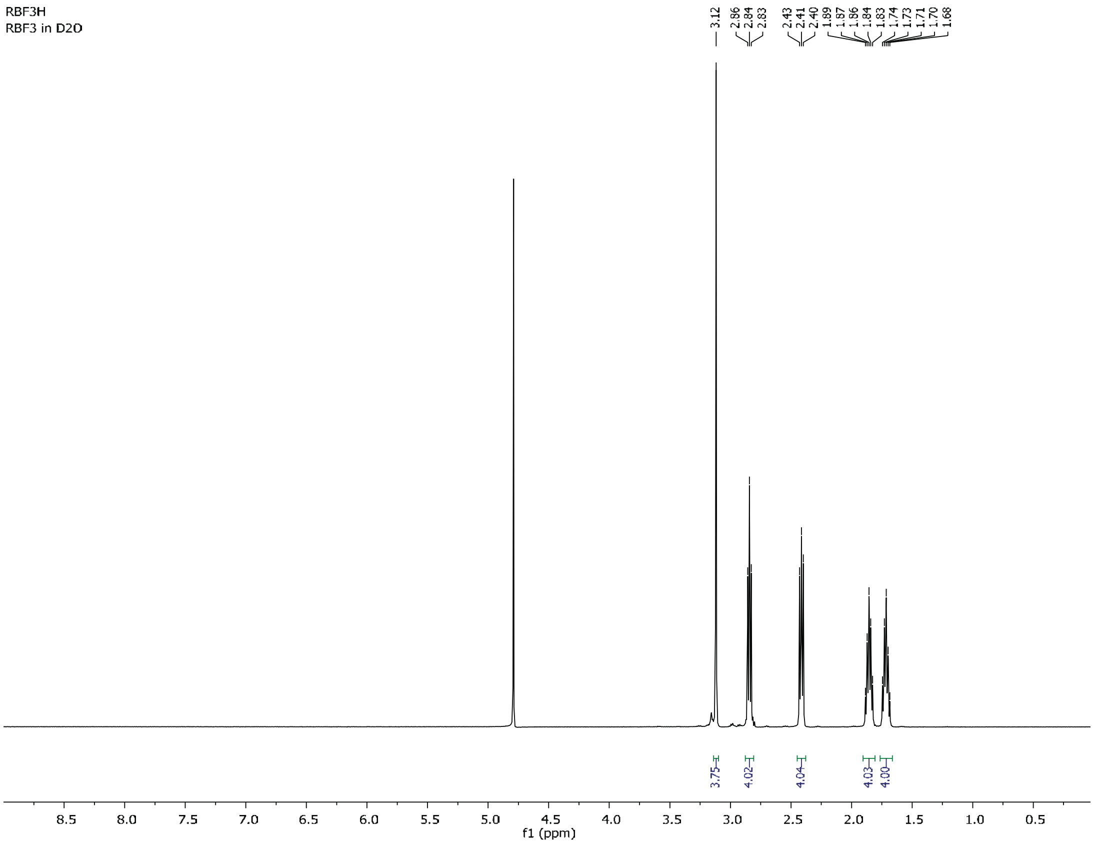

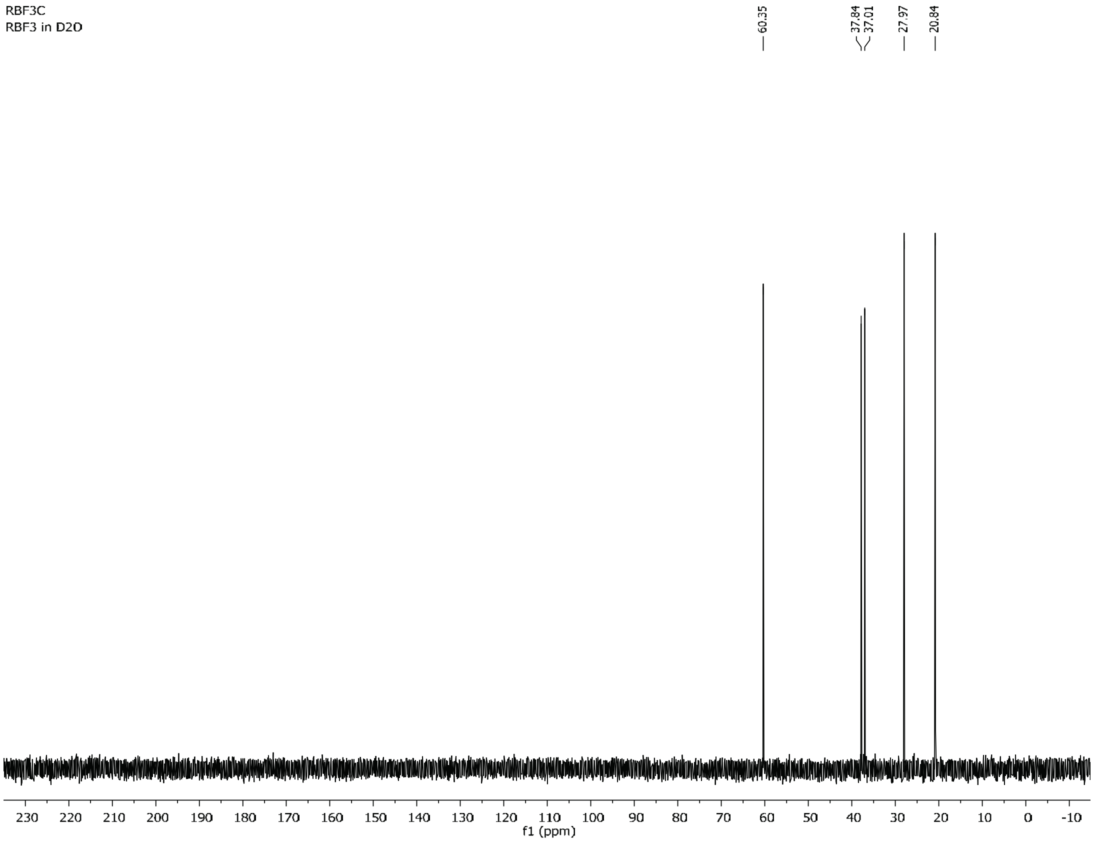

(*Continued*)

Sodium butane-1,4-disulfinate

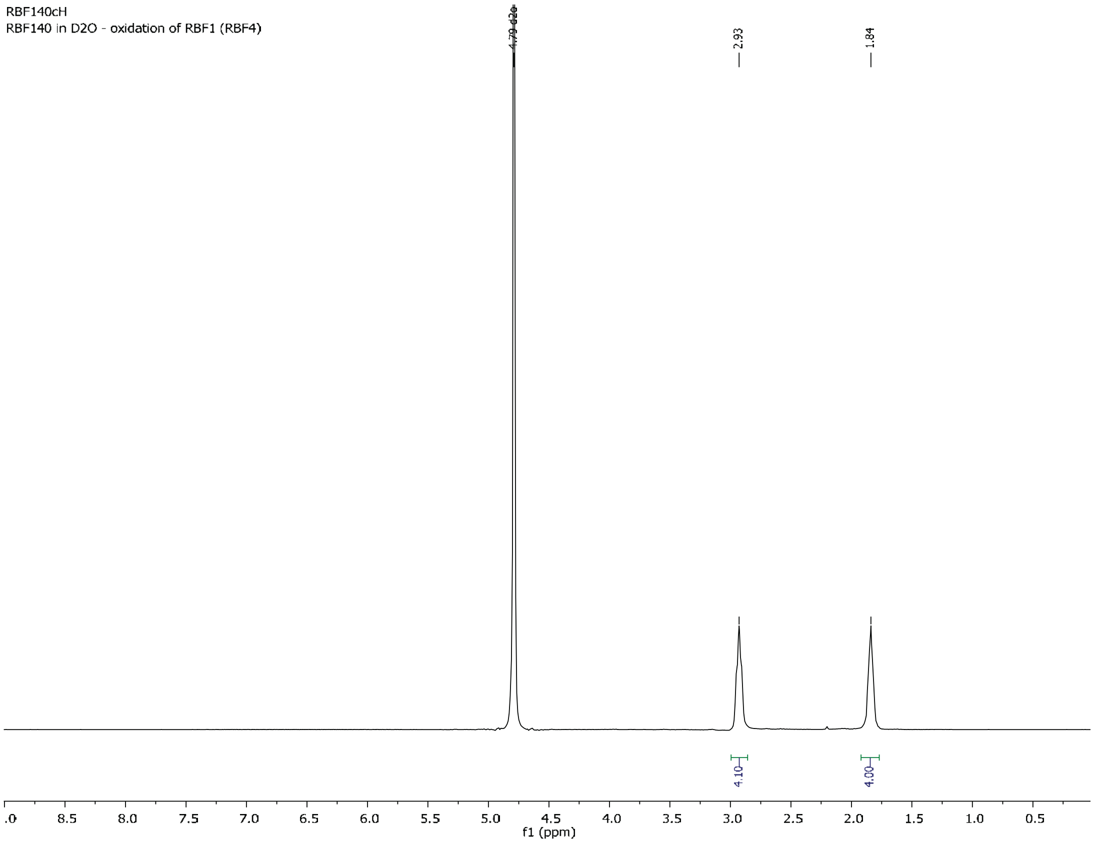

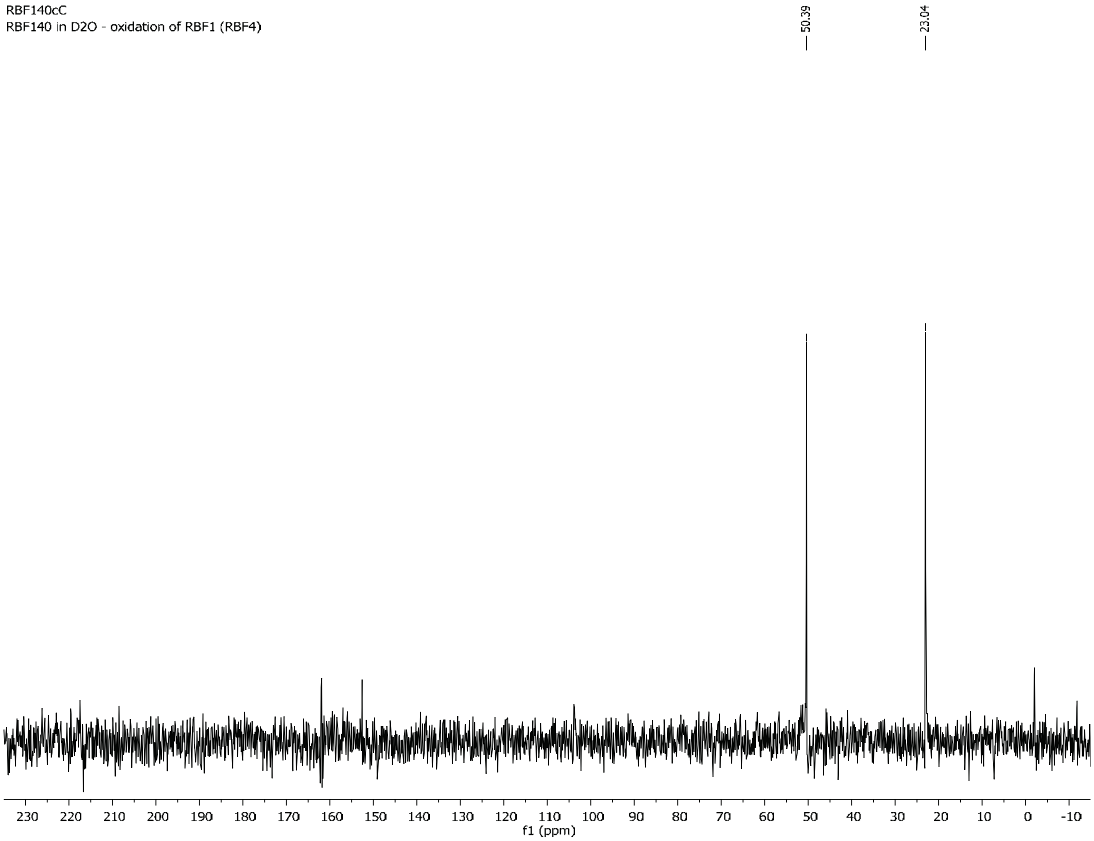

(*Continued*)

Sodium butane-1,4-disulfinate

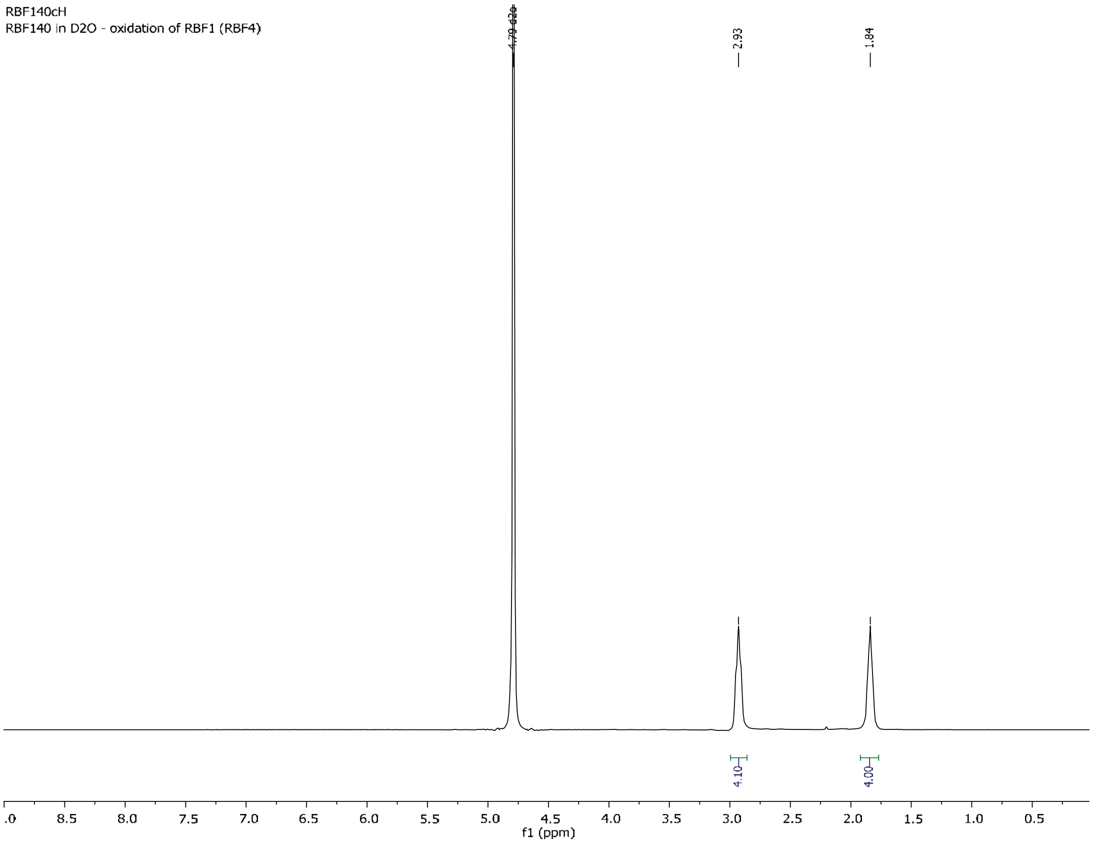

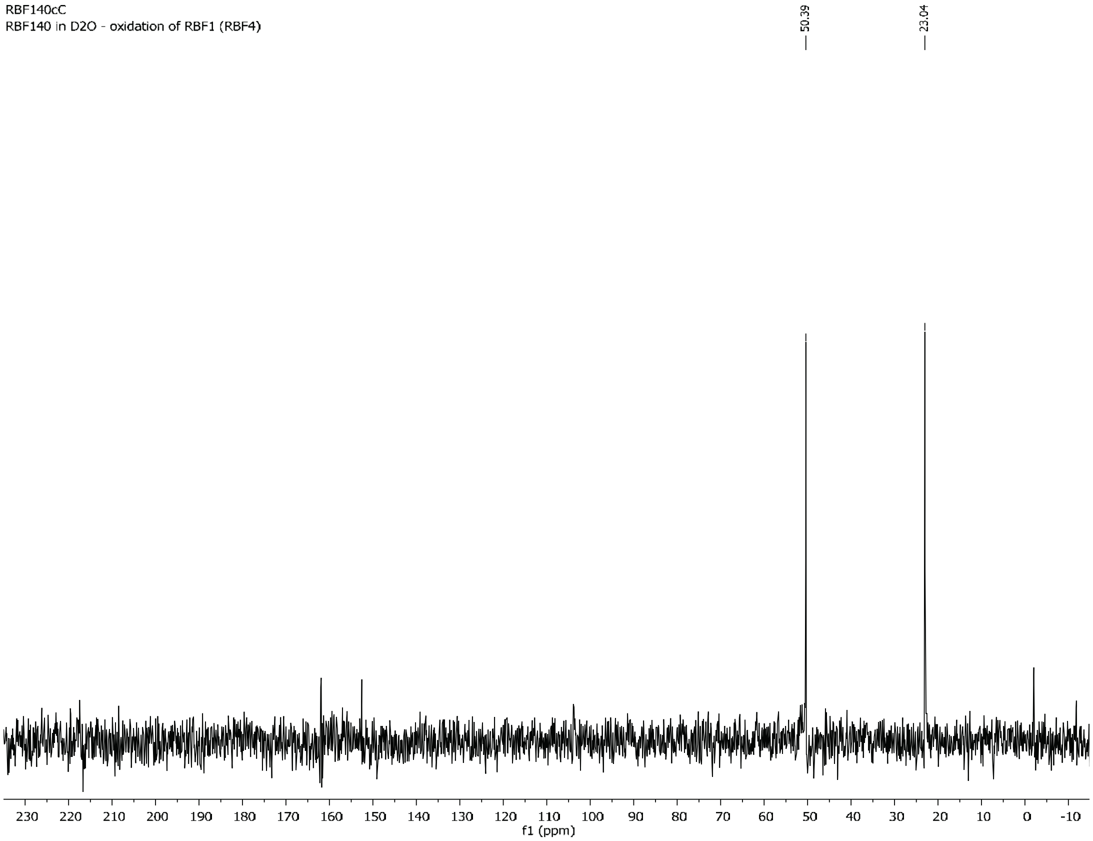

(*Continued*)

RBF4

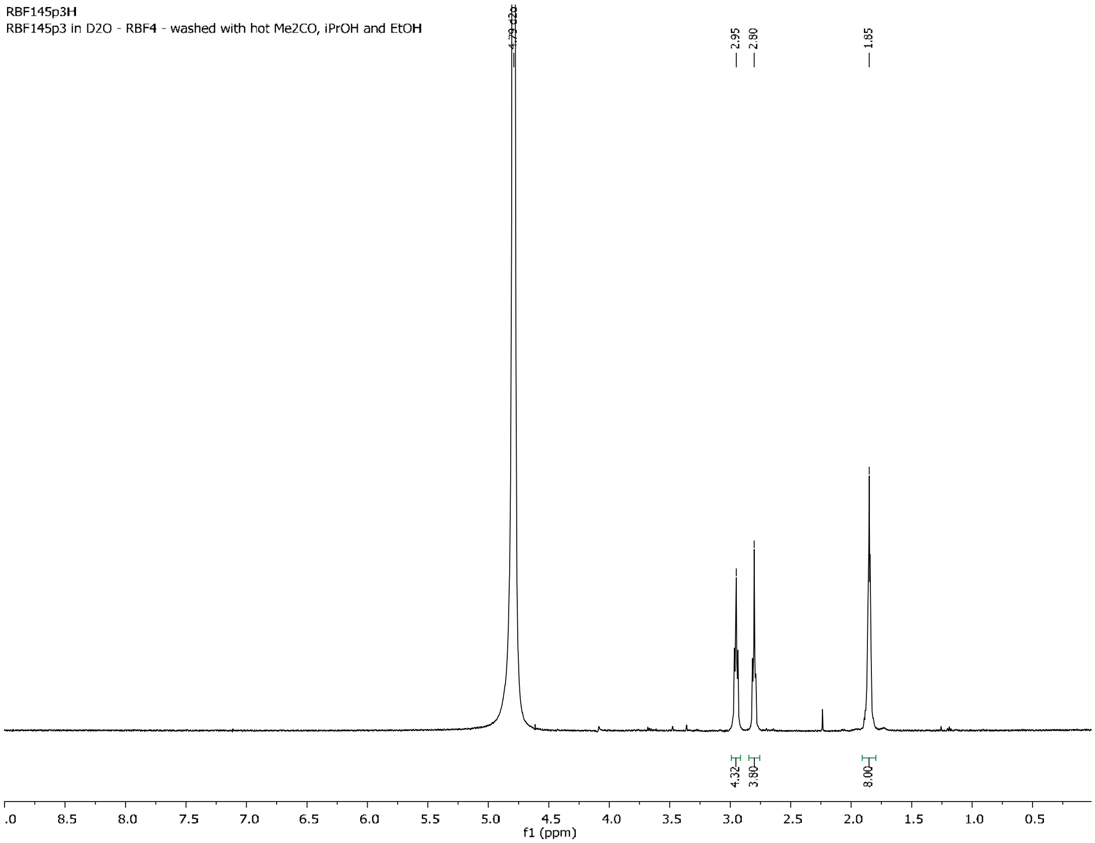

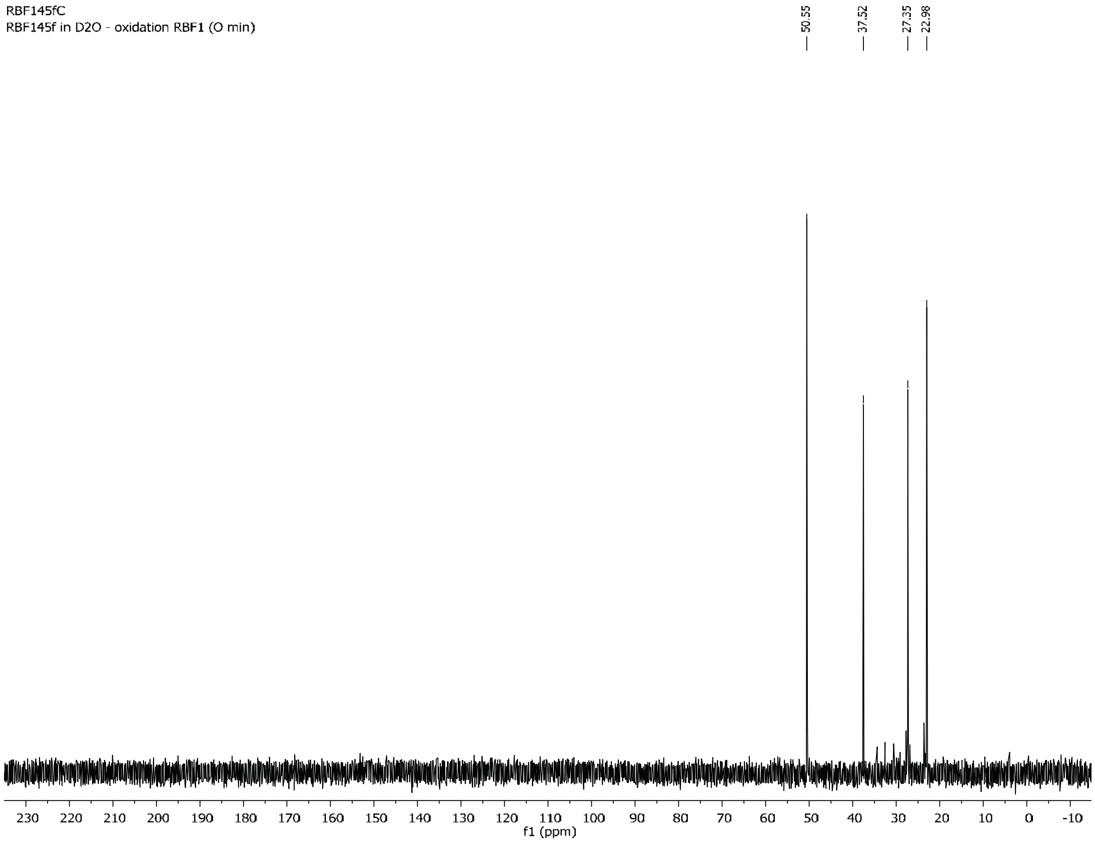

(*Continued*)

RBF6

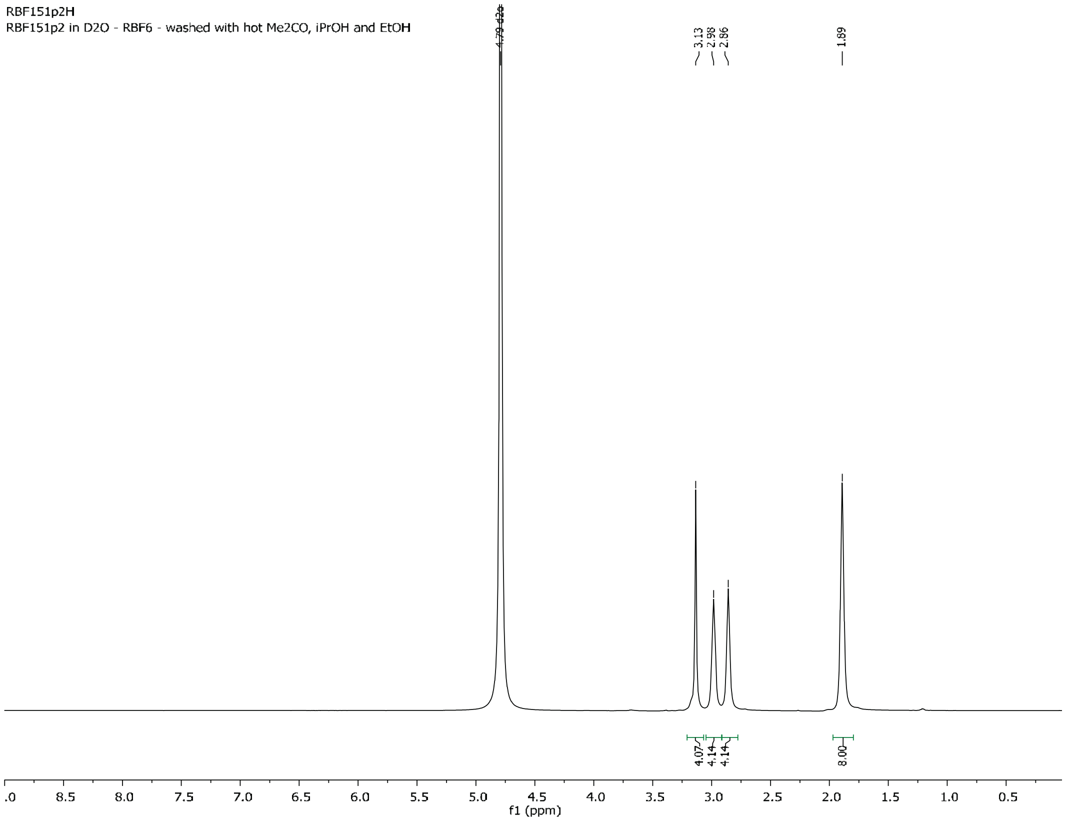
RBF151p2H
RBF151p2 in D2O - RBF6 - washed with hot Me2CO, iPrOH and EtOH
3.13
2.98
2.86
1.89
4.07
4.14
4.14
8.00
f1 (ppm)

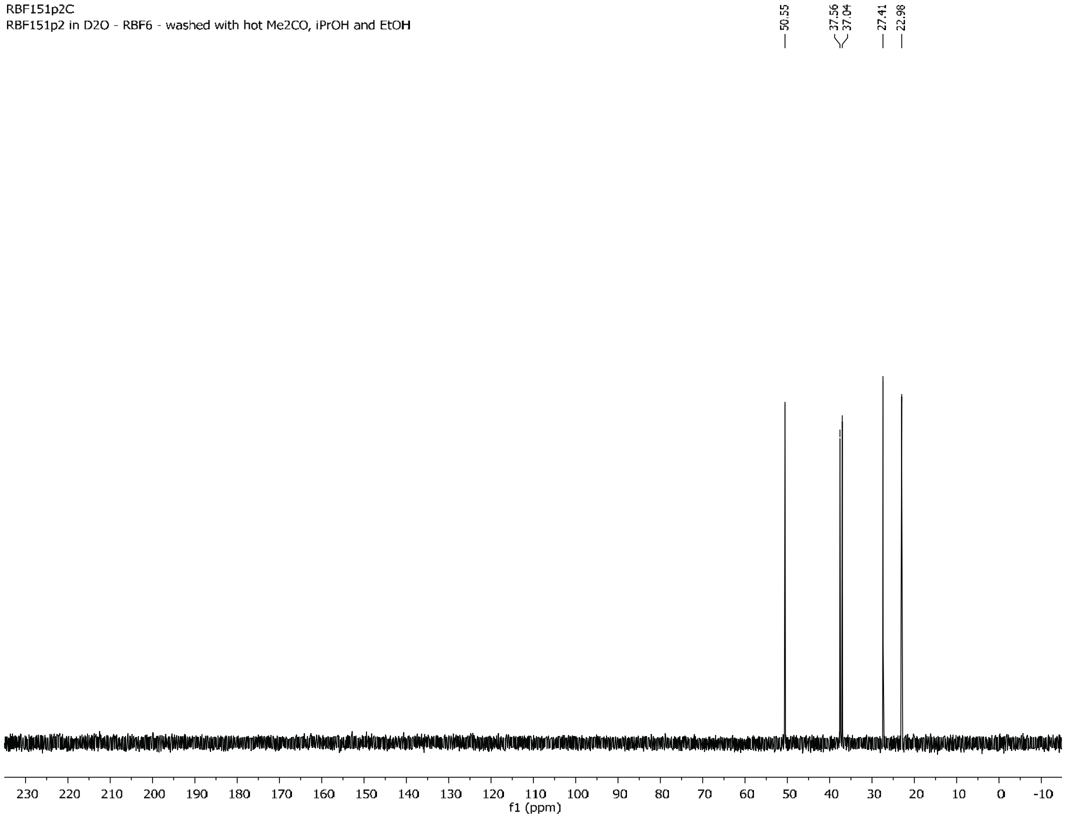
RBF151p2C
RBF151p2 in D2O - RBF6 - washed with hot Me2CO, iPrOH and EtOH
50.55
37.56
37.04
27.41
22.98
f1 (ppm)
